# UK general practice service delivery research priorities: an adapted James Lind Alliance approach

**DOI:** 10.3399/BJGP.2023.0226

**Published:** 2023-12-12

**Authors:** Ruth Abrams, Sharon Blake

**Affiliations:** School of Health Sciences, University of Surrey, Guildford.; School of Health Sciences, University of Surrey, Guildford.

**Keywords:** general practice, qualitative research, research priorities, service delivery, workforce

## Abstract

**Background:**

General practice is in a state of crisis in a number of countries. In the UK, a range of measures have been introduced to address the situation, including innovations such as practice networks, multidisciplinary roles, and digital technologies. However, identifying what still needs fixing could benefit from more evidence, particularly in relation to day-to-day service delivery.

**Aim:**

To identify the general practice workforce’s top 10 research priorities to improve service delivery.

**Design and setting:**

This priority-setting study used an adapted James Lind Alliance methodology and involved staff working in general practice across the UK.

**Method:**

The study comprised four phases: an online qualitative survey issued to the general practice workforce (clinical and non-clinical groups); thematic analysis of free-text responses; generation of indicative research questions; and the undertaking of ranking exercises with responders of the original survey. An online workshop was held with participants at the final stage of prioritisation.

**Results:**

In total, 93 staff completed a survey in Phase 1, from which 20 themes were categorised and developed into research questions. Twenty- two staff responded to the first ranking activity and 11 took part in a second ranking activity to discuss themes that had a tied vote. The final top 10 research priorities were: volume of work; patient behaviour; consultations; employment pay and conditions; workload dumping and care of patients on waiting list; funding; overwhelming pressure; patient health education; complex patient needs; and interfaces with secondary care. However, there was no clear ranking of these 10 priorities; instead, they carried equal weight and were closely interconnected.

**Conclusion:**

Applying a marginal-gains approach, by seeking to explore all 10 priorities simultaneously as opposed to concentrating on one area at a time, may provide more noticeable improvements overall. Systems-based approaches that take account of the marked role that context has may be a particularly useful lens for future research.

## Introduction

General practice, both in the UK and in several other countries worldwide, is referred to as the gatekeeper of health service delivery and referral pathways.^1^ Its core values are patient centred, putting the patient’s wishes, needs, expectations, and preferences at the centre of service delivery.^2^ Escalating demands, compounded by diminishing resources (particularly among the workforce), in general practice are undermining and eroding effective care delivery.^3^ The challenges presented by the COVID-19 pandemic have exacerbated service delivery problems, and further diminished morale and wellbeing in the sector; in addition, staff continue to feel overwhelmed by their workload and experience burnout^4^ — all of which affects staff experience and patient care.^5^ Indeed, patient satisfaction has rapidly declined in recent years.^6^

Although there have been numerous changes to service delivery, including the introduction of additional roles under the Additional Roles Reimbursement Scheme (ARRS), there are still significant recruitment and retention problems that, in themselves, further increase stress among the remaining workforce.^7^^,^^8^ Similar challenges are also faced by other countries, including Australia, Canada, and New Zealand.^9^^–^^11^

The problems facing the general practice workforce have remained the same for almost a decade, if not longer.^12^ These are compound pressures that operate across the entire system and, as such, there are many competing priorities when it comes to addressing them. Priority-setting research can be used to gain consensus about areas with high need, in which engagement in purposeful research could deliver wide-reaching benefit.^13^ It has been suggested that priority-setting research is an essential part of developing a national health research system, as it can guide the allocation of research resources.^14^ However, research agendas to resolve issues are typically directed by government and funding bodies,^15^ with little input from those responsible for delivering services.

In 2004, Black *et al*^16^ set out a healthcare workforce research agenda. Although this was not specific to general practice, it was one of the first to acknowledge the significance of involving the workforce in research, stating that doing so *‘can contribute to resolving existing dilemmas and can provide innovatory ideas for the future’*. Priority-setting research exercises for primary care have been carried out in Scotland^17^ and internationally,^15^ and specifically for general practice in Australia.^18^ The study reported here is, to the authors’ knowledge, the first to look at UK-wide general practice research priorities after COVID-19, specifically from the perspective of the clinical and non-clinical workforce. The aim was to identify the areas of research that should be prioritised by the UK general practice workforce to best meet their identified challenges and proposed solutions to improve service delivery.

**Table:** How this fits in

General practice is recognised as being at crisis point in the UK, not least because of ongoing concerns relating to staff wellbeing, recruitment and retention, and patient access. There are compound pressures across the system with many competing priorities, all of which affect service delivery. This study facilitated input from the general practice workforce to identify the top 10 research priorities for improving service delivery. The findings suggest service delivery priorities need to be addressed simultaneously through a systems-based approach that is both patient and workforce centred.

## Method

The approach to priority setting followed an adapted James Lind Alliance (JLA) prioritisation method.^17^ This was due to its capability in bringing together a diverse group of individuals and adaptability for online use, as outlined in the *James Lind Alliance Guidebook*.^19^ Table 1 provides further details on how the methodology was applied and adapted from the traditional JLA approach. The authors have reported the study in line with Tong *et al*’s^20^ guidelines for reporting priority-setting work. The study had four phases:

- Phases 1 and 2: survey and thematic analysis;
- Phase 3: generation of indicative research questions; and
- Phase 4: ranking and priority setting.

**Table 1.** Traditional processes outlined in the *James Lind Alliance Guidebook*^a^ and their application in this study

**Step**	**Traditional JLA process**	**Application in this study**
Establish a steering group	Often has around 12 members, who are responsible for publicising the initiative, overseeing the checking and collating of uncertainties, and taking the final priorities to research funders.	A steering group of 11 members was established, which included academics, clinicians, and a patient representative. The group helped to guide survey design and dissemination, develop indicative research questions from the data, and disseminate findings. It met twice over a 9-month period and communicated via email.
Gathering uncertainties and searching existing literature for uncertainties	Qualitative process; no maximum or minimum number of responses needed; intended to generate themes and questions; brings together a range of voices; open-ended/free-text responses; open for 2–3 months; requires consent but does not always require ethical approval. Existing literature search includes identifying, for example, research recommendations in literature, such as systematic reviews or protocols.	This step was adapted in two ways: specifically sought workforce voice. This was a small-scale research project and the authors wanted to focus primarily on responses from clinical and non-clinical staff groups — as such, patients/carers were not included; and instead of asking for research questions, the authors designed the survey more broadly to better problematise current understandings. This was done to capture novel areas by encouraging responders to think ‘outside of the box’. To this end, three broad questions were posed. The aim was to make the survey highly accessible without responders needing knowledge about research or research questions.
Data processing	Five steps: download survey data; remove out-of-scope survey submissions; categorise eligible survey submissions; form indicative questions; and verify the uncertainties.	All responses were downloaded into a Microsoft Excel spreadsheet from the Qualtrics survey platform. As the survey was broad, there were no out-of-scope responses and so none needed to be removed. A qualitative, thematic analysis of free-text responses was conducted. This method of analysis was used to allow the authors to best analyse free-text responses and capture nuance across the data. Questions were not requested from responders, so the researchers developed questions, incorporating recommendations from existing literature and steering group involvement — which helped to form and verify the themes. The authors did not search in clinical guidelines but relied on existing systematic reviews to determine research and policy recommendations.
Interim priority setting	Develop an initial list of questions and evidence to present back to the responders for interim prioritisation. This can be issued via another survey, email, or word document, and often contains 60–70 questions. This stage often asks responders to select their top 10 questions.	Process produced a list of 20 themes that were taken back to responders in order to determine which sat within, and without, the top 10. A simple Word document was used for participants to complete ranking.
Final priority setting	Often done face to face, this stage is for responders to discuss the 10 questions. It can involve further ranking or voting. It is about representation and fairness, debate, and consensus. JLA recommends small groups for this work.	An online workshop was conducted to determine the ranking of the top 10. This comprised 11 people but did not involve a trained JLA facilitator.

a The James Lind Alliance Guidebook*^19^ also reports on publishing, taking priorities to funders, and follow-ups. The authors of the study presented here shared the findings with NHS England, NHS Confederation, and the National Institute for Health and Care Research, and also ran a social media campaign to disseminate findings and generate impact (information about which is available from the corresponding author). JLA = James Lind Alliance.*

### Phase 1 and 2: survey and thematic analysis

Clinical and non-clinical staff employed in UK NHS general practice settings were invited to participate in a short qualitative survey. A link to the online survey was circulated on the authors’ behalf through: the NHS Primary Care Staff Network e-bulletin (with a reach of 27 000 employees); professional networks on Twitter/X, including the College of Paramedics and the Queen’s Nursing Institute; and the director’s message in the Royal College of General Practitioners (RCGP) newsletter (which is sent to all members of the RCGP). The survey was open between August and October 2022. Informed consent and basic demographic data were requested, and three open- ended questions that were related to service delivery in general practice were included, namely:

- what is working well? (strengths);
- where do you encounter challenges?; and
- if you were given resources to fix issues relating to service delivery, what would you fix, how, and why? Please list up to five solutions.

Those willing to participate in follow- up activities were invited to leave their names and email addresses. All participants were given an identifier to protect their anonymity. Completed survey responses were downloaded into a Microsoft Excel spreadsheet and analysed thematically, in line with Braun and Clarke’s recommendations.^21^ Responses to the question on strengths were analysed separately but, as responders often referred to solutions when describing challenges, these responses were looked at together. Two researchers reviewed the data and discussed its categorisation to ensure best fit within a defined theme. The strengths data informed the authors’ understanding of what was important to the workforce, but was not included in the prioritisation exercise as that focused on the identified challenges and solutions.

### Phase 3: generation of indicative research questions

A rapid literature search for reviews (not limited in design — for example, systematic, scoping, literature, and narrative) published in English from 2017 was undertaken in November 2022. This was not an exhaustive systematic search, but aimed to give a picture of current research uncertainties relating to the themes identified from Phase 1 survey responses.

A search strategy for the MEDLINE (via Ovid) database was developed and run based on keywords for each of the themes categorised from the survey (Supplementary Box S1). A search for reviews with ‘general practice’, ‘GP’, or ‘primary care’ in the title was also undertaken via Google Scholar, as well as in the Cochrane Library and CINAHL databases. Screening decisions to include a review were based on the relevance of the title to the survey theme. When there was doubt regarding the relevance of an article, the abstract was read.

Key findings and identified research gaps from each included review were extracted into a table and mapped onto each survey theme. If reviews naming any of the identified themes were found, the extracted data then informed the development of the research questions. If multiple reviews suggested a number of research gaps, these were compared with each other (in order to develop a broad understanding of existing uncertainties — for example, around implementation or the need for long-term evaluation) and against the survey data (in order to see what gaps also reflected the identified challenges and proposed solutions). If no reviews naming any of the identified themes were found, the research question was developed from the survey data only and phrased so it would be possible to generate a relevant evidence base for the identified challenges and proposed solutions.

A number of potential research questions were drafted for each theme, then merged and refined in discussion with an expert steering group to formulate one research question that was thought to best reflect the survey data and research gaps.

### Phase 4: ranking and priority setting

A short description of the survey themes and drafted research questions were emailed to all survey responders from Phase 1 who had consented to further participation. In the first round of ranking, responders were invited to rank the themes and research questions from most significant to least significant. Responses were collated into a Microsoft Excel spreadsheet and organised based on the frequency of responses (that is, nominal group technique). This allowed for a refined list of themes and questions, which was then sent to participants in the second round, who were invited to comment on those with tied votes so the final top 10 priorities could be determined.

The top 10 priorities, as ranked through this process, were discussed at an online workshop held in January 2023 and facilitated by one of the researchers. All those who had completed the original survey and expressed an interest in this workshop were invited to attend. This workshop lasted 1 hour and included project background, sharing priorities inside and outside the top 10, sharing reflections, and discussing priority order. Participants who had engaged in prior ranking activities and attended the workshop were thanked for their engagement with a £40 Amazon voucher.

## Results

### Phase 1 and 2: the qualitative survey and thematic analysis

In total, 93 general practice staff completed the survey; most were female (*n* = 59, 63%), White British (*n* = 79, 85%), and aged >40 years (*n* = 74, 80%) (data not shown). The majority of responders worked in clinical roles and had been in service for >10 years (Table 2).

**Table 2.** Characteristics of total sample (*N* = 93)

**Characteristic**	***n* (%)**
**Job title**	
GPs	41 (44)
Nurses	12 (13)
Other direct patient care (for example, paramedics)	15 (16)
Non-clinical (including administrative staff and managers)	25 (27)

**Years in service**	
>10	62 (67)
6–10	12 (13)
2–5	10 (11)
<2	9 (10)

**Self-reported practice size^a^**	
Medium	53 (57)
Large	34 (37)
Small	5 (5)

**Practice setting**	
Urban	57 (61)
Rural	21 (23)
Mixed (semi-rural)	15 (16)

a *One responder worked as a locum so did not provide practice size. Small = 1–3 full-time equivalent (FTE) GPs; medium = 4–6 FTE GPs; and large = >6 FTE GPs.^34^*

Analysis of the challenges and solutions data resulted in the identification of 20 themes. Table 3 provides a summary of the themes, ordered by the number of individual responses prior to any ranking of importance by responders. Overall, the themes with the greatest number of collated responses were:

- routes into general practice;
- volume of work;
- career progression;
- patient behaviour; and
- management of local and network needs.

**Table 3.** Themes identified by total sample (*N* = 93) in Phase 1 survey (prior to ranking)

**Theme**	**Descriptor**	**Responses, *n* (%)**
Routes into general practice	Recruitment of all professions, retention of admin/reception staff	54 (58)
Volume of work	Patient demand, administration, and associated implications on service delivery	36 (39)
Career progression	Need for consistent training, mentoring, and continuous personal development	35 (38)
Patient behaviour	Unrealistic expectations, negative media, abuse, need for sanctions	33 (35)
Management of local and network needs	Utility of primary care networks, effective links with local services, and assessment of local needs	33 (35)
Employment pay and conditions	Better pay, pensions, childcare support	32 (34)
Integration of ARRS	Impact on workload, public acceptance, triaging processes	32 (34)
Funding	Slow payment processes, quantity and fit of targets, lack of inflation adjustment	30 (32)
Digital innovations	Need for integration across services, increased training on systems	27 (29)
Workload dumping and care of patients on waiting list	Additional unfunded work and patient demands while awaiting secondary care falling to general practice	26 (28)
Patient health education	Need for education on self-management for minor illness, who to contact, and training on digital health services	23 (25)
Clarity of aims	Mismatch between workforce priorities (for example, continuity of care) and policy focus on efficiency	22 (24)
Complex patient needs	Lack of best practice and guidance on how to manage multimorbidity	21 (23)
Consultations	How long, how many, and which mode of delivery in what circumstance	21 (23)
Overwhelming pressure	Stress, wellbeing of workforce	21 (23)
Policy involvement	Representation in policymaking and new initiatives	20 (22)
Interface with secondary care	Improved access, clearer pathways, and communication	18 (19)
Supervision of ARRS	Unclear lines of responsibility and match of skills for effective supervision	15 (16)
Physical setting	Adequacy and sustainability of practice buildings	12 (13)
Managing absence	Uncapped cost and challenge to cover staff absence	9 (10)

*ARRS = Additional Roles Reimbursement Scheme.*

### Phase 3: generation of indicative research questions

The rapid literature search resulted in >2500 results, from which 67 reviews were included as relevant to a survey theme (Table 4). Key findings and identified research gaps from each included review are set out in Supplementary Box S2. No reviews were found for the following five themes: volume of work; workload dumping and care of patients on waiting list; supervision of ARRS; physical setting; and managing absence.

**Table 4.** Search results by source

**Database/search engine**	**Results, *n***	**Included reviews, *n***
Ovid MEDLINE	1949	46
Cochrane Library	362	4
CINAHL	273	1
Google Scholar	First 5 pages	16

Twenty research questions were developed in collaboration with the steering group from summaries of the survey themes and identified research gaps per survey theme, as presented in Supplementary Box S3.

### Phase 4: ranking and priority setting

In total, 22 of the 74 responders who had agreed to be contacted again took part in the first round of ranking the identified themes and research questions in order of importance. Eleven responders took part in the second round of ranking, which helped to determine themes with a tied vote. Frequencies were calculated to determine which research questions featured in the top 10 (Table 5).

**Table 5.** Frequency reporting of themes and research questions inside and outside of top 10 in the first round of ranking

**Frequency, Theme**	***n* (%)**
**Inside top 10**	
Volume of work	20 (91)
Patient behaviour	17 (77)
Consultations	16 (73)
Employment pay and conditions	16 (73)
Workload dumping and care of patients on waiting list	16 (73)
Funding	15 (68)
Overwhelming pressure	15 (68)
Patient health education	13 (59)
Complex patient needs^a^	11 (50)
Interface with secondary care^a^	11 (50)

**Outside top 10**	
Digital innovations^a^	11 (50)
Clarity of aims	13 (59)
Physical setting	14 (64)
Policy involvement	15 (68)
Career progression	15 (68)
Supervision of ARRS	15 (68)
Integration of ARRS	15 (68)
Routes into general practice	16 (73)
Management of local and network needs	17 (77)
Managing absence	19 (86)

a *Tied after the first round of ranking.*

*ARRS = Additional Roles Reimbursement Scheme.*

Eleven responders, the majority of whom were GPs, attended the online prioritisation workshop to discuss the final top 10. This group included five responders who had engaged with the study in full and six who had only completed the survey. Although attendees all agreed that the top 10 themes selected were the most significant, they also agreed that it was not possible to rank them in order of importance. As an example, difficulties were experienced in decoupling the following themes: workload dumping and care of patients on waiting list and interface with secondary care; funding and its relationship to other themes; and the volume of work, which was often a consequence of other themes. Workshop attendees determined that if, for example, while researching solutions or interventions to support employment conditions and pay, research into patient behaviour is not also explored simultaneously, the issue of overwhelming pressure and volume of work will likely persist. Similarly, if funding continues for additional specialist clinicians without due attention paid to, for example, patient health education, it is possible that workload issues may persist.

**Figure 1.**
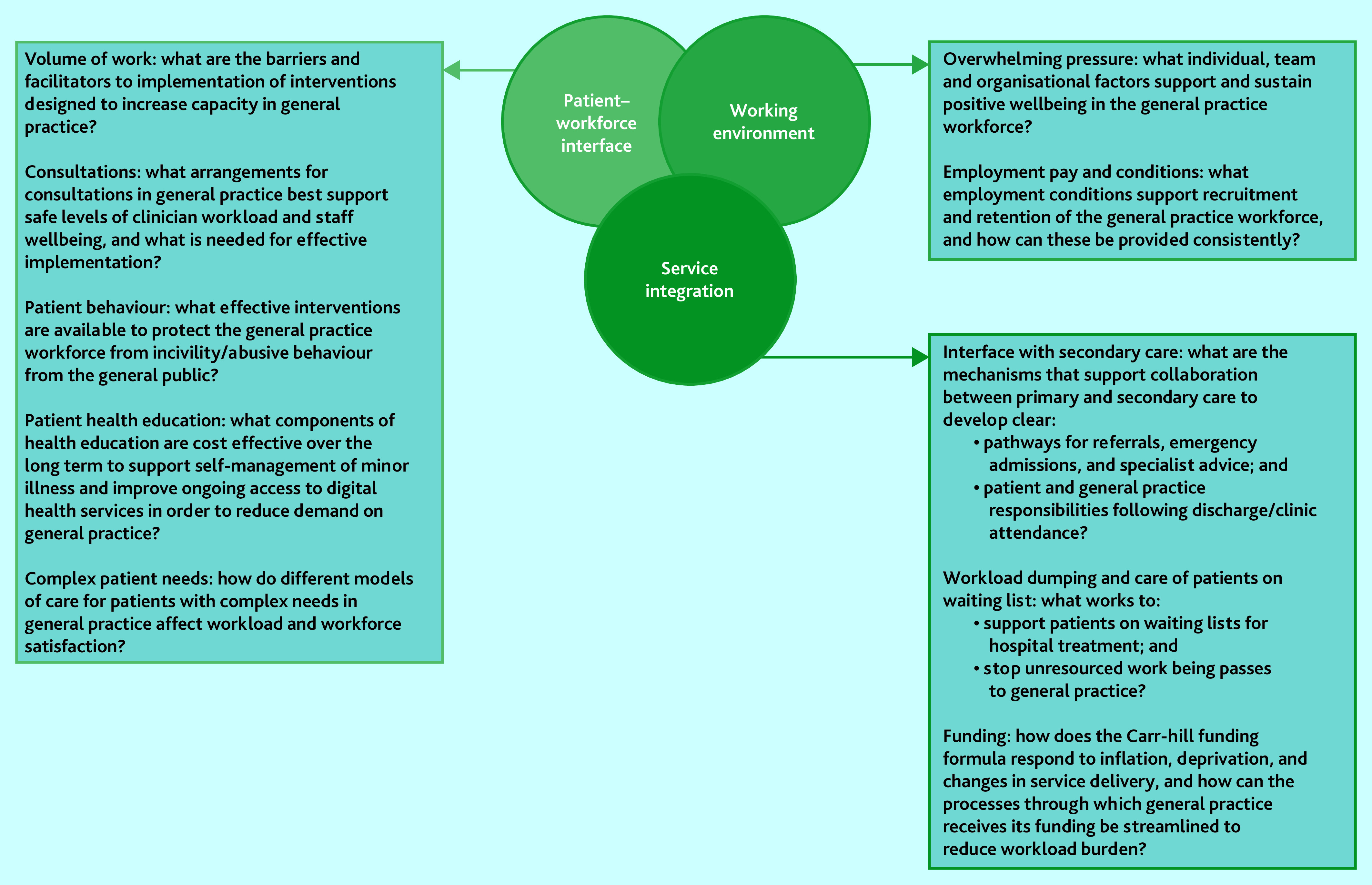
Top 10 research priorities.

Figure 1 presents a visual summary of the overall outcome from the final priority- setting workshop. Depicting priorities as an interconnected Venn diagram was a suggestion provided by workshop attendees. A number of infographics have been produced as a result of study findings (see Supplementary Figures S1–S5).

## Discussion

### Summary

To the authors’ knowledge, this is the first research prioritisation exercise in UK general practice service delivery to be undertaken exclusively with the workforce and include clinical and non-clinical staff groups. Across four phases of work, 10 inter-related areas for improving service delivery were identified as key research priorities, namely: volume of work; patient behaviour; consultations; employment pay and conditions; workload dumping and care of patients on waiting list; funding; overwhelming pressure; patient health education; complex patient needs; and interface with secondary care. Participants determined that these research priorities carry equal need for attention and, as such, any attempt to research one component of service delivery in isolation is unlikely to lead to long-term change.

### Strengths and limitations

This study drew on a well-recognised methodological approach to undertaking research priority-setting work, with few adaptations. For the purposes of this study, general practice was considered to be a universal service, but the authors recognise that there are multiple services delivered in general practice by a range of staff. There was a notable absence of certain groups — for example, physician associates — and a low response rate; this could point to the challenge of securing survey participants when the given workforce is under stress. In addition, given the small-scale nature of the project and limited resources, the patient voice was not included in the study. Existing research has suggested differences in priorities between workforce and patient groups, who may be more likely to focus on access.^17^ By including a range of clinical and non-clinical staff groups, however, this research provides a greater understanding of what the workforce sees as important issues in need of further research. Research priorities were based on staff experiences, which is a recognised way of contributing ideas to a JLA study, without explicitly asking for research questions.^19^

The rapid literature search supported a broad understanding of general practice research; however, it was not undertaken on a systematic basis and, by only looking for keywords in titles, reviews are likely to have been missed. As only one member of the research team made decisions regarding inclusion, it is also impossible to rule out the risk of bias in the study selection.

The initial survey indicated that routes into general practice, volume of work, career progression, patient behaviour, and how general practice is managed appeared of potential importance. However, only two of these areas were selected as part of the final top 10 (volume of work and patient behaviour). The differences between responses from the wider survey and the top 10 list identified by the prioritisation exercise point to a need to keep in mind the influence that ‘those in the room’ have on what is determined a priority or not;^22^ this is a critique of priority-setting studies more broadly.

Although this study explores UK general practice, it may have international relevance for countries that share a similar general practice design and are also in a time of crisis in the sector.

### Comparison with existing literature

Many of the themes identified by the general practice workforce in this study share parallels with previous empirical studies. For example, in their mixed- methods study exploring why GPs leave practice early, Doran *et al*^7^ suggested that reasons included funding and overwhelming pressure. Research by Riley *et al*^23^ demonstrated the emotional demands placed on clinical staff when dealing with abusive patient behaviour, indicating this as a source of stress — which was typified by the survey responders of the study reported here. Issues relating to the volume of workload, indicating the saturation point at which general practice is operating, was determined by Hobbs *et al*^24^ as far back as 2016, and the work by Naqvi *et al*^25^ highlighted the difficulties in working across care interfaces.

When asked to prioritise themes, attendees appeared to focus more on challenges and solutions that were conceived as falling outside of the control of the general practice workforce, such as interface with secondary care, managing complex patient needs, and patient health education; this is notable because not having control over day-to-day practice has been recognised as having a negative effect on stress.^26^^–^^28^ Along with the use of technology, these same three research areas (health education, complexity of needs, and interface with secondary care) were also identified by others as priorities,^15^^,^^17^^,^^18^ suggesting a need to maintain such focus. However, the study presented here also identified new themes, such as volume of work, overwhelming pressure, patient behaviour, and employment pay and conditions. This highlights that existing research may fall short in considering the impact of service delivery on the workforce, and understanding which areas the workforce considers significant. Although the rapid review showed that some survey themes (for example, complex patient needs) are receiving substantial research attention, extant research does not typically focus on, or include examination of, the workforce perspective of the impact that service design and delivery has on staff (for example, in relation to workload and job satisfaction). Other survey themes that participants deemed important to address (for example, workload dumping and care of patients on waiting list, employment conditions, physical setting, and managing absence) have yet to attract research attention.

### Implications for research and practice

The rapid literature search undertaken in this study suggests that general practice research is typically narrow (focused on specific patient groups or professions), unless undertaken outside of academia by organisations — such as the King’s Fund — which appear to lead on research to inform general practice policy. Future research now needs to undertake more comprehensive evidence mapping across the 10 identified priorities to extend the rapid literature review.

Future research also needs to be commissioned to explore the 10 identified priorities simultaneously. Doing so requires approaching general practice as a system that is designed as a whole,^29^ which means drawing on modes of thought and delivery that are capable of addressing contextual factors that span across levels.^30^ This raises important questions for researchers and policymakers as to how to best tackle the 10 priorities identified in this study. To this end, applying a marginal-gains approach, by seeking to explore all 10 priorities simultaneously, may provide more noticeable improvements overall, as opposed to concentrating on one area at a time.^31^^,^^32^ This may also have wider application beyond primary care to broader service delivery and associated compound pressures across the entire system.

Reflecting the finding that the study sample prioritised challenges that fall outside of general practice control, multidisciplinary research that can creatively explore or group research priorities together is needed. To this end, certain methodologies might be more useful, including systems-based approaches^30^^,^^31^ and approaches that take account of the significant role of context, for example, realist.^33^

A commitment is needed from researchers looking at general practice to consider the impact of their work on patients, service delivery, *and* the workforce. In particular, paying attention to the mutuality of benefit for staff and patient care may help to transform current vicious cycles into virtuous ones. This needs to be done concurrently with exploring the broader narrative about general practice, including what ‘good’ looks like, and what general practice means — and does — in the contemporary world.
